# Post-Traumatic Growth during COVID-19: The Role of Perceived Social Support, Personality, and Coping Strategies

**DOI:** 10.3390/healthcare10020224

**Published:** 2022-01-25

**Authors:** Chu-Si Xie, Yunhwan Kim

**Affiliations:** Department of Psychology, Faculty of Social Science, Lund University, 22100 Lund, Sweden; yunhwan.kim@psy.lu.se

**Keywords:** post-traumatic growth, social support, personality traits, coping strategies, COVID-19 pandemic

## Abstract

Although many studies on mental health have been conducted among various populations during the COVID-19 pandemic, few studies have focused on post-traumatic growth (PTG) in the general population. The current study aimed to explore whether perceived social support, personality, and coping strategies are associated with PTG in the COVID-19 pandemic period. The study also investigated whether coping strategies mediate the relations between perceived social support, personality, and PTG. A total of 181 participants (*M*_age_ = 24) completed the self-report questionnaire online, which was distributed via various online channels, mainly in China and Sweden. The relations between the study variables were examined with correlation analyses and a multiple mediation analysis. Results showed that more than half of the participants (60.8%) reported experiences of PTG during the pandemic. Additionally, perceived social support, personality traits (extraversion, emotional stability, agreeableness, and conscientiousness) and coping strategies (problem-focused coping, emotion-focused coping, and social support coping) were positively correlated with PTG. In addition, coping strategies (problem-focused coping, emotion-focused coping, and avoidance coping) mediated the relations between perceived social support, personality traits and PTG. Theoretical and practical implications of this study are discussed, concluding that the findings of this study have the potential to guide intervention efforts to promote positive change during the pandemic.

## 1. Introduction

Since the advent of the COVID-19 pandemic, its adverse consequences on mental health have been increasingly elaborated on [1,2]. Although research has also recently started paying attention to positive aspects of pandemic experiences [3], still little is known about the pandemic’s potential positive psychological outcomes and to what extent and how this may trigger post-traumatic growth (PTG) [4]. The current study aims to fill these research gaps.

PTG is defined as the subjective experience of positive psychological change reported by an individual as a result of struggling with trauma [5,6]. A meta-analysis indicated that 50–60% of people exposed to potentially traumatic events are likely to experience some degree of PTG [7], which leaves a possibility that PTG might have occurred during the COVID-19 pandemic.

The psychological process growing out of adversity is challenging and may require personal, interpersonal and sociocultural characteristics [6,8]. The current study examined factors that might influence PTG during the context of COVID-19, including situational characteristics (social support) and personal characteristics (personality traits and coping strategies). The findings are expected to show whether and how the general population experienced PTG during the COVID-19 pandemic and, therefore, to guide policymakers and practitioners in developing meaningful practices and interventions for future pandemic events. Identifying predictors and mechanisms of PTG is imperative to safeguarding people from the harmful effects of COVID-19 and promote PTG.

### 1.1. Post-Traumatic Growth in the Context of COVID-19

PTG involves changes in five areas: finding new possibilities in life, enhanced relationships with others, subjective feelings of personal empowerment, greater appreciation of life and spiritual development [6]. It indicates that people with COVID-19-related PTG may report that they care more about family members and friends’ feelings or take more time to relax and adopt a healthier lifestyle. In addition, they may learn to be grateful for their health, family, friends, and daily lives. Indeed, a recent scholarly discourse also highlighted that, while affected people may struggle with mental issues, changes in their lifestyle can simultaneously facilitate PTG; they are able to engage in many leisure activities that they rarely had time to do before the COVID-19 pandemic, including reading in peace, learning new skills, or enjoying meals with their family [9]. Since the impact of COVID-19 is rippling through all aspects of society, including financial setbacks, social media influences, and personal and community restrictions, some researchers consider COVID-19 to be a new type of mass trauma. However, in the literature, little is known about PTG in the context of the COVID-19 pandemic, which deserves further investigation [10,11].

It is widely believed that PTG occurs after devastating physical or psychological trauma. Many studies have focused on people with cancer [12], HIV [13], and those who have experienced natural disasters [14]. Thus, it is not surprising that, during the COVID-19 pandemic, PTG-related studies focused primarily on the healthcare population, which is a high-risk population [3,15,16]. However, the reactions associated with the current pandemic situation may not be limited to the diagnosis of serious illnesses, but are also a by-product of our ongoing awareness of our vulnerability to infection and the multiple medical, psychological, and social consequences of the disease [4,17]. The threat from COVID-19 is constant, invisible, and pervasive compared to other common traumatic events [18]. The threats affect the general population and are not limited to high-risk groups [17]. Thus, it is also necessary to pay attention to the PTG of general populations during the COVID-19 pandemic [19].

### 1.2. Social Support and Post-Traumatic Growth

The Functional Description Model of Tedeschi and Calhoun [6] explains the development of PTG. Tedeschi et al. believed that traumatized individuals have their core beliefs challenged and experience emotional struggles, and that growing out of adversity requires personal characteristics and sociocultural characteristics. Specifically, an individual’s personal characteristics (e.g., personality) influences how they experience the traumatic event. Social support (e.g., material or emotional comfort and support from the outside world) and positive coping processes (e.g., detachment from unachievable goals and unsustainable beliefs; reduction of emotional distress through effective emotion regulation strategies) help the individual to move on from their struggle with trauma. Therefore, PTG is achieved through the interplay of personal characteristics (e.g., personality), sociocultural influences (e.g., social support), and cognitive processes (e.g., coping strategies), as well as other factors [6,8].

Social support is a common predictor included in many previous studies on PTG [20,21]. Social support has been widely interpreted in two ways: perceived social support and received social support [22]. Perceived social support concerns the subjective evaluation of how individuals perceive friends, family members as available to provide material, and psychological and overall support during times of need, whereas received support relates to the actual quantity of support received [22]. The relationship between perceived social support and PTG was explored in this study. It is widely believed that high perceived social support predicts high PTG [21,23,24,25,26]. Given that several studies have reported that people during COVID-19 often feel isolated and alienated and have difficulty accessing social support [27], there is a need to further clarify the role of perceived social support within PTG during the COVID-19 pandemic.

### 1.3. Personality Traits and Post-Traumatic Growth

Different empirical studies provide varying degrees of evidence to support the positive relationship between personality traits and PTG, including extraversion [6,26,28], agreeableness [29], conscientiousness [26,28,30], and optimism [31,32]. The findings on emotional stability are inconsistent; some studies did not show any significant relationship with PTG [33], while others showed a positive relationship [28]. The findings on openness to experience are also inconsistent. Some studies did not reveal any significant relationship with PTG [34,35], while others demonstrated a positive relationship [6,30]. Few studies have explored the relationship between personality traits and personal growth in the context of the pandemic [30], especially during the COVID-19 pandemic, so further research is needed to explore the relationship between personality and PTG.

### 1.4. The Role of Coping Strategies on Post-Traumatic Growth

Coping strategies have been widely examined as one of the factors in the PTG process [36]. Different coping strategies are associated with differences in psychological adjustment [3,37]. First, most studies have shown that problem-focused coping, such as active coping and planning, are positive predictors of PTG [38,39]. Second, research has found that emotion-focused coping, such as acceptance and positive reframing, can facilitate meaning-making and encourage PTG [20,38,40]. Third, few studies have directly explored the relationship between social support coping and PTG. Some relevant research suggests that people who actively seek social support to help manage stressors may also be more effective at coping with traumatic stressors [41], which is supported by a post-earthquake PTG study which reported that people who were more willing to seek help had higher PTG [42]. Fourth, an increasing number of studies have explored the relationship between avoidance-coping and PTG [38,43,44]. Some studies have shown that avoidance-coping is a maladaptive coping strategy [38,39,45]. However, emerging research has indicated that avoidance-coping decreases symptom distress and increases PTG [43,46,47]. Avoidant coping may play an adaptive role some time after a traumatic event [43,46]. Furthermore, coping may be flexible across situations, with strategies that are effective in specific events and not effective in others [48]. Thus, it is worthwhile to explore the relationship between coping strategies and PTG during the COVID-19 pandemic.

Previous studies have also shown that coping strategies play a mediating role between other situational/personal factors and PTG [20,42,49,50]. First, coping strategies supported by empirical research (problem-focused coping, emotion-focused coping and social support coping) were found to mediate the relationship between perceived social support and PTG [20,42,50,51]. That is, individuals who perceive strong social support are likely to be more capable of using appropriate coping strategies, and therefore more likely to report PTG [20]. Second, the relationship between personality traits and PTG is mediated through various coping processes [49]. Researchers suggested that adaptive coping mediated the relationship between the five-factor personality and PTG [52]. Research has shown that individuals with high extraversion scores adopted higher levels of problem-focused coping and social support coping. Furthermore, research has found that problem-focused coping mediates the relationship between extraversion and PTG [34]. At the same time, individuals with high conscientiousness, agreeableness and emotional stability presented with higher levels of problem-focused coping and emotion-focused coping [53,54]. A study supported the mediating role of problem-focused coping in the relationship between conscientiousness and positive emotions [55]. What is lacking in the literature is about how four coping strategies mediate the relationship between perceived social support, personality and PTG during the COVID-19 pandemic in particular.

### 1.5. The Present Study

This study aims to examine the role of perceived social support, personality, and coping strategies within PTG. In addition, this study investigates the mediation effect of coping strategies in the relation between perceived social support, personality and PTG. The hypotheses of the current study are as follows:

**Hypotheses** **1.1** **(H1.1).** *Perceived social support is positively correlated with PTG.*

**Hypotheses** **1.2** **(H1.2).** *Extraversion, agreeableness, and conscientiousness are positively correlated with PTG.*

**Hypotheses** **1.3** **(H1.3).** *Problem-focused, emotion-focused, and social support coping are positively associated with PTG.*

**Hypotheses** **2.1** **(H2.1).** *Coping strategies (problem-focused, emotion-focused, and social support coping) mediate the relationship between perceived social support and PTG.*

**Hypotheses** **2.2** **(H2.2).** *Coping strategies (problem-focused, emotion-focused, and social support coping) mediate the relationship between personality traits (extraversion, agreeableness, and conscientiousness) and PTG.*

**Figure 1:**
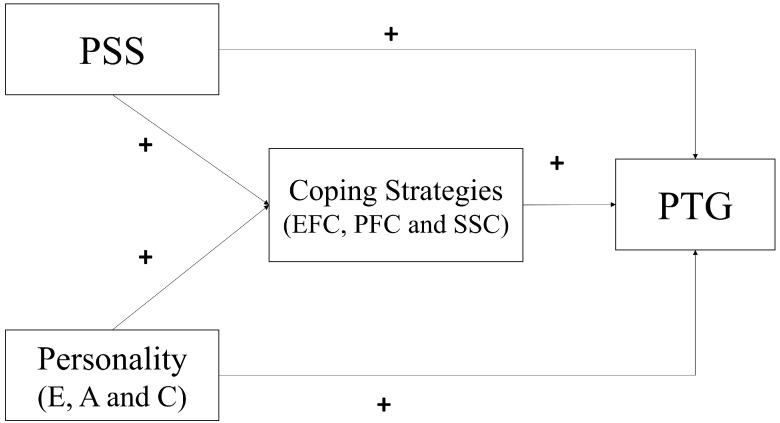
Hypothesized path model for PTG. Note. The figure describes the hypothesized paths of personality, PSS, coping strategies and PTG. PSS = Perceived Social Support; E = Extraversion, A = Agreeableness, C = Conscientiousness, PFC = Problem-focused Coping, EFC = Emotion-focused Coping, SSC = Social Support Coping, PTG = Post-Traumatic Growth.

Figure 1 shows the hypothesized model. No hypotheses were generated for openness, emotional stability, and avoidance coping due to inconsistent findings in the literature and, therefore, these are exploratory in nature.

## 2. Materials and Methods

### 2.1. Procedures and Participants

The convenience sampling method was used in this cross-sectional quantitative study. The data were collected through Qualtrics, an Internet-based survey platform, which provides multiple fraud detection options to increase data quality, including enabling multiple submission prevention and detecting duplicated responses. According to ExpertReview, a data quality check feature in the Qualtrics platform, the responses of this study were considered reliable and there were no duplicate respondents.

After an online survey was constructed, a pilot test was first conducted with 11 respondents to assess the clarity and the response time of the questionnaires (about 10 min), and data from the pilot study were not included in the final data analysis. Then the link to the formal survey was distributed from 25th April to 5th May 2021 via a social media (Facebook), an online discussion forum (Reddit), and instant messaging apps (WhatsApp and WeChat). The inclusion criteria for participants in this study were: (1) an age range of 18 years and older, and (2) the ability to use English proficiently.

Of the 213 participants who took part in the surveys, 32 were excluded because the respondent did not complete the survey. A chi-square analysis showed that there were no significant differences in the demographic characteristics between those who completed the survey and those who did not do so. Therefore, 181 surveys were used for further analyses, resulting in an 85.0% rate of completion. The sample was predominantly female (70.2%) with a mean age of 24.68 years (*SD* = 5.40).

### 2.2. Measures

Based on the research purposes, we chose questionnaires that were widely known and used in the literature on PTG and in the literature on stress and coping, including the Posttraumatic Growth Inventory-Short (PTGI-S) [19,44], Multidimensional Scale of Perceived Social Support (MSPSS) [20,51], The Ten-Item Personality Inventory (TIPI) [56], and Brief COPE Questionnaire [29]. In addition to these measures, a demographic information form was administered to the participants, which includes information on their gender, age, employment status, country of residence, and living situation during COVID-19. Given the uneven distribution of the pandemic around the world (7 January 2022, https://coronavirus.jhu.edu/map.html) and the different policies in each country (e.g., lockdown or not), which has been shown to be strongly related to people’s mental health [27], the country of residence was chosen for this study instead of the country of origin. The questions were modified to specifically ask respondents to indicate their situation during the COVID-19 pandemic.

#### 2.2.1. The Post-Traumatic Growth Inventory-Short (PTGI-S)

The PTGI-S [6] was used to measure the degree of positive change experienced during the COVID-19 pandemic, which has excellent psychometric properties (Cronbach’s α = 0.91). The five subscales are (1) relating to others, (2) new possibilities, (3) personal strength, (4) spiritual change, and (5) appreciation of life. Higher scores indicate more significant degrees of change. Questions use a 6-point Likert scale from (1) no change to (6) a high degree of change. Scores at the 60th percentile or higher (≥32) indicate potential personal growth.

#### 2.2.2. The Multidimensional Scale of Perceived Social Support (MSPSS)

The MSPSS [57] was used to measure perceived social support during the COVID-19 pandemic. The scale included 12 items describing perceived support from family, friends, and significant others (e.g., “I can talk about my problems with my family”, “My friends really try to help me”). Items were ranked on a 7-point Likert scale, ranging from (1) strongly disagree to (7) strongly agree. Higher scores indicate a higher level of perceived social support. In the present study, the Cronbach’s alpha value was 0.80.

#### 2.2.3. The Ten-Item Personality Inventory (TIPI)

The TIPI [58] is a self-report measure designed to assess the Big Five personality traits [59]. The ten items are rated on a 7-point Likert scale ranging from (1) Disagree strongly to (7) Agree strongly, and grouped into five dimensions. Cronbach’s alpha values were calculated for extraversion (Cronbach’s α = 0.75), agreeableness (Cronbach’s α = 0.42), conscientiousness (Cronbach’s α = 0.58), emotional stability (Cronbach’s α = 0.56), and openness (Cronbach’s α = 0.40).

Although a part of the subscales presented lower alpha values than conventionally accepted values, it was not totally unexpected. Indeed, in the study by Gosling, Rentfrow and Swann [58] where TIPI was developed and validated, the reported Cronbach’s alphas were 0.68 for extraversion, 0.40 for agreeableness, 0.50 for conscientiousness, 0.73 for emotional stability, and 0.45 for openness to experience, which were similar to the present study. Gosling, Rentfrow and Swann [58] concluded that TIPI achieved an adequate level in each of the criteria evaluated: convergent and discriminant validity, test–retest reliability, and patterns of external correlates. In addition, due to the large number of questions measured in this study, the use of the TIPI was strategically chosen in order not to increase the burden of participants in filling out the questionnaires, but to improve the quality of the data received.

#### 2.2.4. Brief COPE Questionnaire

The Brief COPE Questionnaire [60] consisted of 28 items that assessed how participants coped with the stress they faced during the COVID-19 pandemic. The Brief COPE consists of 14 conceptually distinct subscales. Four-point scales were used to measure the extent to which the participants were coping with stress associated with the COVID-19 pandemic, ranging from (1) I haven’t been doing this at all to (4) I have been doing this a lot. Based on a four-factor structure [61], we grouped the coping strategies to measure problem-focused coping (active coping and planning; α = 0.84), avoidant coping (behavioral disengagement and denial; α = 0.77), social support coping (emotional support, instrumental support, and venting; α = 0.81), and emotion-focused coping (positive reframing, acceptance; α = 0.76).

### 2.3. Statistical Analysis

All demographic factors were summarized using frequencies and percentages. The relationships between the key variables were assessed using Pearson correlation analysis. Next, mediation analyses were conducted using the AMOS version 26 software. All path coefficients were estimated using the maximum likelihood method based on the multivariate normality of observable variables, and their significance was assessed using bootstrapping with 2000 replications. The coefficient of determination (*R*^2^) was computed to assess how well the model explains the outcome. All tests were two-tailed, and a *p*-value of less than 0.05 was statistically significant.

In the path analysis, model fit was tested through chi-square (X^2^), the X^2^*/df* index (acceptable: <5; good: <2), the comparative fit index (CFI, acceptable: between 0.90 and 0.95, good: >0.95), the goodness-of-fit index (GFI, acceptable: between 0.90 and 0.95, good: >0.95), the root mean square error of approximation (RMSEA, acceptable: <0.10, good: <0.06), and the standardized root mean square residual (SRMSR, acceptable: <0.10, good: <0.08) detecting whether the model fit is good or acceptable [62].

A post hoc power analysis was performed to evaluate the power of the statistical analysis [63]. Given the final sample size (*n* = 181), alpha level at 0.05, and an effect size of 0.46, we were able to achieve a post hoc power larger than 0.99, which is greater than the conventional adequacy standard of 0.80.

## 3. Results

The descriptive statistics for PTG and its subdomains, demographic characteristics of participants, and the relationship between demographic factors and PTG are shown in Table 1. The sample was predominantly female (70.2%) and students (81.2%), with a mean age of 24.68 years (*SD* = 5.40). The participants were mainly from China (81.8%). A total of 84.0% of the participants reported living with others, and more than half of the participants reported living in China (61.3%) during the COVID-19 pandemic, followed by 22.1% living in Sweden. For PTG, there was no gender difference in this study (*p* = 0.92). 

The average PTG score was 33.41 (*SD* = 10.88), and 60.8% participants with scores at the 32 points or higher demonstrated personal growth during COVID-19. As Table 1 shows, amongst PTG subdomains, personal strength was scored the highest by participants (*M* = 7.02; *SD* = 2.65) along with relating to others (*M* = 7.00; *SD* = 2.53) and appreciation of life (*M* = 6.81; *SD* = 2.53). 

### 3.1. Correlations

Means, standard deviations, and correlations for the key variables are presented in Table 2. As expected, correlational analyses indicated that perceived social support (*r* = 0.30, *p* < 0.001), extraversion (*r* = 0.26, *p* < 0.001), agreeableness (*r* =0.16, *p* < 0.05), conscientiousness (*r* = 0.18, *p* < 0.05), and emotional stability (*r* = 0.30, *p* < 0.001) had a significant positive correlation with PTG, which supported hypotheses 1.1 and 1.2. There were positive correlations between PTG and using problem-focused coping (*r* = 0.46, *p* < 0.001), emotion-focused coping (*r* = 0.54, *p* < 0.001), and social support coping (*r* = 0.28, *p* < 0.001), which supported hypothesis 1.3. No significant correlation was found between PTG with openness (*r* = 0.07, *p* = 0.35) and avoidance coping (*r* = 0.13, *p* = 0.09).

### 3.2. Mediation Models

**Figure 2:**
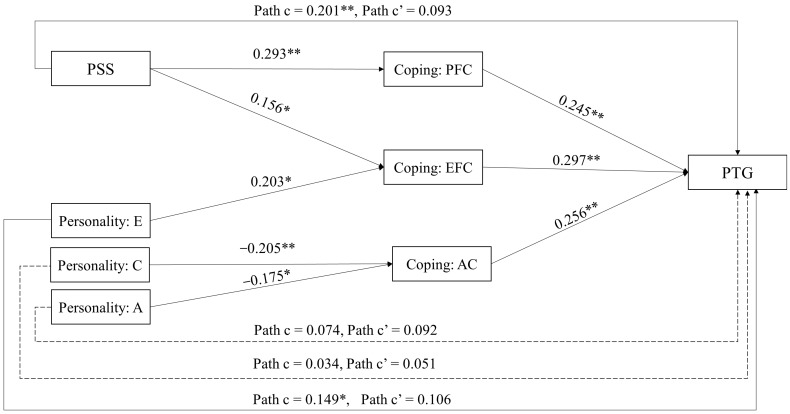
Direct, and total effects in multiple mediation model. Note. Path c represents total effect, path c’ represents direct effect. PTG = Post-Traumatic Growth, PSS = Perceived Social Support, E = Extraversion, A = Agreeableness, C = Conscientiousness, PFC = Problem-focused Coping, EFC = Emotion-focused Coping, AC = Avoidance Coping. Only significant mediation effects are shown in the Figure, along with the relevant results of the complete model. For the complete results which include the results from the variables included in the analysis which, however, were not presented above due to brevity (i.e., emotional stability, openness, and social support coping), please refer to Appendix A. ** = *p* < 0.01, * = *p* < 0.05.

A multiple mediation model was constructed to test if the participants’ coping strategies mediated between their perceived social support and post-traumatic growth and between their personality traits and post-traumatic growth. The goodness-of-fit assessment of the path model showed that X^2^ (20) = 34.25, *p* = 0.025, X^2^*/df* = 1.71, RMSEA  =  0.06, CFI  =  0.98, GFI =  0.98, and SRMR  =  0.03, indicating the good model fit of the path model. The model explained 46% of the variance of PTG. Table 3 showed the significant mediation effects, and corresponding paths were shown in Figure 2. Only the significant indirect effects and the relevant direct effects are presented in the Table and Figure for readability. For example, results for social support coping are not presented in the Figure because no main effect and mediating effect of social support coping was found, which was inconsistent with the hypothesis. In addition, openness could not affect PTG directly or indirectly through any coping strategy. However, note that the complete results are available in Table A1 of Appendix A.

From the results of the total effect in Figure 2, perceived social support (*β* = 0.201, *p* < 0.01) and extraversion (*β* = 0.149, *p* < 0.05) could effectively predict PTG, and among the coping strategies, problem-focused coping (*β* = 0.245, *p* < 0.01), emotion-focused coping (*β* = 0.297, *p* < 0.01), and avoidance coping (*β* = 0.256, *p* < 0.01) were positive predictors of PTG. As Table 3 shows, the results support hypothesis 2.1 that problem-focused coping mediates the relationship between perceived social support and PTG (*β* = 0.072, *p* < 0.001, 95% CI [0.023, 0.098]), and emotion-focused coping mediates the relationship between perceived social support and PTG (*β* = 0.046, *p* < 0.05, 95% CI [0.007, 0.042]). The results partially support hypothesis 2.2, that emotion-focused coping mediates the relationship between extraversion and PTG (*β* = 0.06, *p* < 0.01, 95% CI [0.093, 0.478]). Avoidance coping mediated the relationship between agreeableness and PTG (*β* = −0.045, *p* < 0.05, 95% CI [−0.368, −0.049]), and between conscientiousness and PTG (*β* = −0.052, *p* < 0.01, 95% CI [−0.433, −0.090]).

## 4. Discussion

The purpose of the current study was to identify factors that might predict PTG in response to the COVID-19 pandemic in an effort to find a measure to enhance it. More than half of the participants showed PTG, which confirmed that pandemics can bring about positive outcomes [7], along with traumatic and negative ones [64]. In addition, the current study identified some mechanisms that lie behind post-traumatic growth during the pandemic.

In line with previous research [20,26,65], perceived social support positively influenced PTG. One possible explanation is that high perceived social support can provide a sense of having a safe environment, emphasize feelings of belonging, serve as a buffer against stress, provide new meaning, and generate more positive perceptions that endorse growth [6,8]. Besides, according to the Functional Description Model [6], the mediating effect of coping strategies also explains the relationship between perceived social support and PTG. Perceived social support facilitates cognitive processes, and people use coping strategies to identify positive meanings for personal growth [24]. The participants with high perceived social support in our study used more problem-focused coping and emotion-focused coping and presented higher levels of PTG, supporting the above claims.

The positive effect of extraversion on PTG was observed, consistent with previous studies [21,26,28]. People high in extraversion are fun-loving, active, and optimistic [19], so one possible explanation for the result is that people high in extraversion are more likely to express their emotions and expose themselves to others, which promotes PTG in interpersonal interactions [21]. Another possible explanation is also based on how extroverted persons are more optimistic. Specifically, optimistic individuals tend to focus on the most important matters and reject unachievable goals that are inconsistent with reality, which could lead to PTG [21,66]. Furthermore, people with high extroversion are more likely to use more support-seeking, problem-solving, and cognitive restructuring strategies, and achieve higher levels of PTG [53]; the mediating effects of emotion-focused coping between extraversion and PTG in our study support this explanation.

Positive correlations between conscientiousness and agreeableness and PTG were found, and further analysis revealed that their relationships were mediated by avoidance coping. This suggests that people with low agreeableness or low conscientiousness adopt more avoidance coping, and avoidance coping in turn alleviates stress and promotes growth to some extent [43]. People with low conscientiousness are characterized by low self-regulation, impulsiveness, and undiscipline [59]. People with low agreeableness tend to be manipulative, ruthless, uncooperative, and irritable [67]. Given this backdrop, it is not surprising that people with low conscientiousness or low agreeableness did not proactively deal with traumatic events, but rather take an avoidant approach [53]. It should be noted that some mediating effects were small in the present study and may need to be further validated. 

The positive correlation between emotional stability and PTG contradicts some studies [5,33,34], yet it supports others [28]. Our finding indicates that emotionally stable people are able to better handle stressful situations and regulate their emotions. This implies that emotionally stable people may benefit from their emotional stability which protects them from negative effects of stressful events, such as the COVID-19 pandemic.

The most robust predictors of PTG were the use of problem-focused coping, emotion-focused coping and avoidance coping. Coping is accepted as “a transactional process between individuals, the context, and post-trauma outcome” [52] (p. 326). When faced with a stressful event during COVID-19, the participants in our study who adopted problem-focused coping tended to take a positive coping attitude and make a detailed plan to work through the problem to eliminate the stress, which is vital to maintain one’s quality of life [38]. Besides, people who adopt emotion-focused coping are more inclined to solve problems emotionally by changing their cognitive processes, reconstructing what happened, and/or adopting an attitude of acceptance to make peace with the situation [38]. These strategies seemed to help the participants in our study reform their views on the COVID-19 pandemic, and consequently facilitate them to overcome COVID-19-related problems [68].

Avoidance coping as a positive predictor of PTG in this study contradicts some studies [38,39,45] but supports the results of others [43,44,46,47]. One explanation is that coping is situation-specific, and is cultural and context-dependent [46]. It has been suggested that avoidance-coping plays an adaptive role as a common response in the early post-traumatic period and promotes PTG [21,46,69]. In addition, an avoidant reaction alleviated the patient’s anxiety to a greater extent in a Chinese study [70]. Another explanation is that appropriate avoidance may be beneficial in reducing symptoms of distress and improving patients’ quality of life [69]. In the current global pandemic, the general population faces a stressful event beyond one’s own control at an individual level. People cannot eradicate the virus, but are exposed to panic and anxiety. Perhaps the only thing people can do in such a situation is to find a way to extricate themselves from the anxiety of the stressful event. Thus, it is possible to get positive change through active distraction [71].

Moreover, researchers have proposed the Janus Face Two-Component model [72], which conceptualizes PTG as both an outcome of trauma and a strategy to relieve emotional distress. The model includes two components of PTG: a functional side and an illusory side. The functional side refers to the positive psychological outcome that results from the trauma, as illustrated in the Tedeschi and Calhoun [6] model. In contrast, the illusory side refers to treating PTG as a strategy or process, rather than an outcome. Some researchers have argued that if avoidance strategies promote PTG, it indicates the illusory side of PTG [43]. The significant effect of avoidance-coping in the present study is in line with this argument. 

Social support coping was positively correlated with PTG, but further analysis revealed that social support coping did not significantly predict PTG and there was no mediating effect of social support coping. One explanation is that during the COVID-19 pandemic, people’s access to social support was objectively hindered. Participants are not able to entirely rely on their preferred coping strategies for dealing with stress (e.g., socializing with other people, seeking social support) [10]. Another explanation is that people who use social support coping perceive more social support rather than vice versa. Thus, perceived social support might mediate the relationship between social support coping and PTG, rather than social support coping acting as a mediator between perceived social support and PTG. Further investigations are needed to test this speculation.

### 4.1. Implication

To our knowledge, this is the first study that was conducted to examine the associations between social support, personality, coping, and post-traumatic growth specifically in the context of the COVID-19 pandemic. Previous studies have not yet touched upon this kind of pandemic, which interrupts or prevents people from public socialization globally. The current study expanded the scope of research into this new phenomenon. The findings of this study suggest that our previous knowledge on post-traumatic growth, which were generated based on other categories of traumatic experiences, such as cancer, war, and natural disasters, has great potential to be generalizable to a new type of mass trauma brought about by COVID-19. The results highlight the vital role of personal characteristics (e.g., personality traits and coping strategies) and external resources (e.g., social support) in explaining the PTG, and revealed indirect routes that might contribute to PTG during the COVID-19 pandemic. The findings of this study not only supplement the growing body of literature on the psychological consequences of the COVID-19 pandemic, but also contribute to the literature by exploring the mediating role of coping strategies in the relationship between personality and PTC, and between perceived social support and PTG.

The findings have implications for clinical practice, and even informing strategies for public health authorities. It is essential for clinicians to teach individuals how to utilize different coping strategies that are carefully tailored to their level of perceived social support and personality in order to successfully handle their pandemic-related concerns and thoughts. This is in line with the suggestion by Trobst [73] that intervention procedures may be more effective if tailored for individuals based on personality dispositions. For example, individuals scoring low on conscientiousness may benefit from the skill and active distraction, while the outgoing extrovert may respond best to offers of emotional support. In addition, a severe shortage of resources could influence people’s access to social support through conventional means, such as social support coping, during the COVID-19 pandemic. It implies the need for a variety of timely psychological support, including telemedicine and informal support groups [74].

### 4.2. Limitations

The present study has some shortcomings that should be acknowledged. The first limitation was related to the research design. The current study adopted a cross-sectional design, which accompanies two drawbacks. First, a cross-sectional design limits our ability to make causal inferences about the observed relationships. Therefore, it is noteworthy that our discussions and conclusions about the directions between the variables were only based on the theoretical conceptions, and were not substantiated by empirical test; thus, it is impossible to conclude from our study that the use of coping strategies lead to PTG. Second, although PTGI-S is psychometrically valid, without a pre-COVID-19 psychological profile, self-reported data from a single time-point may be subject to possible recall bias and social desirability. In order to obtain as much information as possible about the positive changes related to COVID-19 and to partly address the above-mentioned weaknesses, we specifically emphasized that the questions in the survey concerned situations during the COVID-19 period to the participants. However, we cannot be entirely sure that the data represent the actual growth of the participants. Hence, a longitudinal study is needed to enable more rigorous comparisons across time-points.

The second limitation was related to the sampling method. Although the post hoc power analysis showed that the current study obtained an adequate level of power, undeniably, the small sample size and convenient sampling method limited the representativeness of the sample in this study. Lack of randomization or representative samples may have led to a selection effect. That is, it might have been the case where people with high PTG were more willing to participate in the survey, while people with low PTG, who supposedly suffer from mental health issues, were more reluctant to respond to the online survey. The survey was conducted via the Internet, where its users tend to be younger; this might explain the overrepresentation of young people in our sample. The lack of older people in the sample may be particularly noteworthy given that they are more vulnerable to many risks driven by the COVID-19 pandemic. Additionally, most of the participants in this study were living in China, although a separate test only with the participants residing in China (*n* = 111) yielded similar results to those obtained from the entire sample. These may all lead to limitations in the generalizability of the findings to other similar populations.

The third limitation was the low Cronbach’s alpha of some personality traits in the current study which we specified earlier in the methodology section. Again, given the research context, TIPS was a strategic choice, and TIPS is considered as adequate despite its inherent limitation of lower-than-usual Cronbach’s alpha values due to its small number of items [58]. However, it is undeniable that some more nuanced details might be captured if a more sophisticated and reliable personality measure is used.

The fourth limitation was that psychopathological conditions were not considered in this study. There were two reasons why psychopathology was not considered. First, unlike many other studies that have examined the negative effect of the COVID-19 pandemic on mental health issues [75], our aim was to clarify the relationship between PTG and the relevant factors. Second, it has been suggested that the general population may experience less fear and psychopathology due to lower morbidity and mortality from COVID-19 than high-risk populations, such as health-care workers [75]. However, the absence of information on the psychopathology of the participants indeed limited our potential to either control for or investigate the potential roles that psychopathology might have played in the relationship between PTG and related factors, which deserves further exploration.

The last limitation was that we did not consider the participants’ country of origin, which may have an impact on PTG. An individual’s religiosity and spirituality are shaped by the context in which they were grown, and many studies have found a clear relationship between religiosity and PTG [76]. Additionally, research has indicated that individuals’ cognitive strategies are influenced by their cultural background (e.g., religiosity and spirituality) [77]. Although we had our reasons to collect information on the participants’ country of residence during the COVID-19 pandemic, the lack of information on the participants’ country of origin undeniably limits our interpretation of the relationship between PTG and related factors.

### 4.3. Further Research

This study also provides directions for future research. First, future studies can adopt longitudinal designs to fully disentangle the underlying causality among the variables. Second, in addition to assessment by self-reporting, further research should consider clinical and observational data, or means of neuroscience, to fully understand the mechanisms behind PTG. Third, future efforts can be made to ensure to recruit more representative samples, including various age groups and different cultures, to reduce any biases that can be derived from selection effects. This would help to fill the gap that the current study leaves to the literature on the PTG of the general population. Fourth, cultural differences in PTG are also worthy of further investigation. Kashyap and Hussain [78] stated that individuals’ cognitive strategies and growth processes are influenced by their culture, so it is crucial to research PTG within the framework of the individual’s culture. In this study, the majority of participants were from China. Therefore, our findings illustrate the mechanisms of PTG in the context of the COVID-19 pandemic, mainly from a collectivist culture [79]. PTG in other cultural contexts (e.g., individualism) needs further study. Finally, as mentioned in the discussion, the relationship between avoidance-coping and PTG, and the possibility that perceived social support may mediate the relationship between social support coping and PTG, deserves further investigation.

## 5. Conclusions

In summary, the current study aimed to identify factors and mechanisms that might enhance PTG during the COVID-19 pandemic. Most people experience post-traumatic growth during the COVID-19 pandemic period. As expected, our findings indicate that perceived social support, some personality traits (extraversion, agreeableness, conscientiousness, and emotional stability), and some coping strategies (problem-focused coping, emotion-focused coping, and social support coping) are positively correlated with PTG during the pandemic. In addition, the association between perceived social support, personality factors (extraversion, agreeableness, and conscientiousness), and PTG are mediated by coping strategies (problem-focused coping, emotion-focused coping, and avoidance coping).

## Figures and Tables

**Table 1:** Descriptive statistics of participants and PTG (N = 181).

				PTG		
Variables	Frequency	%	Range	*Mean*	*SD*	*p*
**Total PTG**			10–60	33.41	10.88	
Relating to Others			2–12	7.00	2.53	
New Possibilities			2–12	6.75	2.53	
Personal Strength			2–12	7.02	2.65	
Spiritual Change			2–12	5.84	2.6	
Appreciation of Life			2–12	6.81	2.53	
PTG ≥ 32	110	60.80%				
PTG < 32	71	39.20%				
**Age**			19–47	24.68	5.40	
**Gender**						0.92
Male	54	28.8%		33.3	11.70	
Female	127	70.2%		33.5	10.60	
**Employment status**						0.91
Employed	46	25.4%		33.3	12.60	
Unemployed	135	74.6%		33.5	10.30	
**Living situation**						0.12
Living alone	29	16.0%		30.6	8.95	
Living with other	152	84.0%		34.0	11.20	
**Student or not**						0.16
Yes	147	81.2%		35.4	11.70	
No	34	18.8%		32.9	9.78	
**Country of residence during COVID-19**						
China	111	61.3%		32.5	10.40	
Sweden	40	22.1%		31.5	8.28	
Other countries	30	16.6%		41.1	12.80	

Note. PTG = Post-Traumatic Growth.

**Table 2:** Correlations and descriptive statistics of key variables.

		1	2	3	4	5	6	7	8	9	10	11
1	PTG	1										
2	PSS	0.30 ***	1									
3	E	0.26 ***	0.21 **	1								
4	A	0.16 *	0.27 ***	−0.02	1							
5	C	0.18 *	0.23 **	0.12	0.30 ***	1						
6	N	0.30 ***	0.15 *	0.43 ***	0.25 ***	0.36 ***	1					
7	O	0.07	0.28 ***	0.25 ***	0.28 ***	0.29 ***	0.20 **	1				
8	PFC	0.46 ***	0.33 ***	0.14	0.13	0.21 **	0.23 **	0.15 *	1			
9	EFC	0.54 ***	0.27 ***	0.29 ***	0.20 **	0.19 **	0.25 ***	0.23 **	0.46 ***	1		
10	AC	0.13	−0.17 *	−0.14	−0.28 ***	−0.32 ***	−0.25 ***	−0.17 *	−0.09	0.05	1	
11	SSC	0.28 ***	0.37 ***	−0.04	−0.02	−0.06	−0.22 **	−0.02	0.40 ***	0.38 ***	0.23 **	1
	*Mean*	33.41	58.83	7.98	8.51	8.56	8.15	8.94	11.99	20.50	7.57	16.96
	*SD*	10.88	15.08	2.80	2.69	2.55	2.62	2.24	2.83	4.05	2.74	4.23

Note. PTG = Post-Traumatic Growth, PSS = Perceived Social Support, E = Extraversion, A = Agreeableness, C = Conscientiousness, N = Emotional Stability, O = Openness, PFC = Problem-focused Coping, EFC = Emotion-focused Coping, AC = Avoidance Coping, SSC = Social Support Coping. *** = *p* < 0.001, ** = *p* < 0.01, * = *p* < 0.05.

**Table 3:** Significant mediation effect for PTG.

	95% Confidence Level		Standardized Estimate
Indirect Path	Lower	Upper	
PSS --> PFC --> PTG	0.023	0.098	0.072 ***
PSS --> EFC --> PTG	0.007	0.042	0.046 *
E --> EFC --> PTG	0.093	0.478	0.060 **
A --> AC --> PTG	−0.368	−0.049	−0.045 *
C --> AC --> PTG	−0.433	−0.090	−0.052 *

Note. PTG = Post-Traumatic Growth, PSS = Perceived Social Support, E = Extraversion, A = Agreeableness, C = Conscientiousness, PFC = Problem-focused Coping, EFC = Emotion-focused Coping, AC = Avoidance Coping. Only significant mediation effects are shown in the table, For the complete results, please refer to Appendix A. *** = *p* < 0.001, ** = *p* < 0.01, * = *p* < 0.05.

## Data Availability

The data that support the findings of this study are available from the corresponding author on reasonable request.

## Appendix A

**Table A1:** Multiple mediation model, total, direct, and indirect effects.

	95% Confidence Level		Standardized Estimate
Path	Lower	Upper	
**Total effect (c)**			
PSS → PTG	0.075	0.316	0.201 **
E → PTG	0.024	0.280	0.149 *
C → PTG	−0.096	0.154	0.034
A → PTG	−0.066	0.202	0.074
O → PTG	−0.232	0.057	−0.091
N → PTG	0.072	0.357	0.215 *
PFC → PTG	0.120	0.382	0.245 **
EFC → PTG	0.170	0.428	0.297 **
AC → PTG	0.147	0.354	0.256 **
SSC → PTG	−0.114	0.127	0.002
Age → PTG	−0.134	0.137	0.002
Living status → PTG	0.071	0.289	0.182 **
Employment situation → PTG	−0.161	0.088	−0.034
Living country → PTG	−0.248	0.036	−0.108
Gender → PTG	−0.063	0.142	0.039
**Direct effect**			
PSS → PTG	−0.018	0.199	0.093
PSS → EFC	0.022	0.273	0.156 *
PSS → PFC	0.157	0.422	0.293 **
PSS → SSC	0.325	0.577	0.453 **
PSS → AC	−0.184	0.107	−0.004
E → PTG	−0.012	0.219	0.106
E → EFC	0.077	0.338	0.203 *
E → PFC	−0.144	0.134	−0.008
E → SSC	−0.145	0.128	−0.002
E → AC	−0.192	0.071	−0.058
N → PTG	0.074	0.296	0.180 **
N → EFC	−0.097	0.235	0.082
N → PFC	−0.008	0.331	0.165
N → SSC	−0.400	−0.101	−0.251 **
N → AC	−0.273	0.048	−0.113
C → PTG	−0.059	0.154	0.051
C → EFC	−0.088	0.188	0.051
C → PFC	−0.083	0.247	0.080
C → SSC	−0.163	0.109	−0.032
C → AC	−0.317	−0.085	−0.205 **
A → PTG	−0.035	0.202	0.092
A → EFC	−0.012	0.238	0.110
A → PFC	−0.152	0.103	−0.021
A → SSC	−0.184	0.068	−0.053
A →AC	−0.301	−0.037	−0.175 *
O →PTG	−0.229	−0.001	−0.114
O → EFC	−0.065	0.207	0.069
O → PFC	−0.140	0.186	0.021
O → SSC	−0.200	0.062	−0.071
O → AC	−0.149	0.135	−0.009
EFC → PTG	0.170	0.428	0.297 **
PFC → PTG	0.120	0.382	0.245 **
AC → PTG	0.147	0.335	0.256 **
SSC → PTG	−0.114	0.127	0.002
Age → PTG	−0.134	0.137	0.002
Living situation → PTG	0.071	0.289	0.182 **
Employment status → PTG	−0.161	0.088	−0.034
Living country → PTG	−0.248	0.036	−0.108
Gender → PTG	−0.063	0.124	0.039
**Indirect effect**			
PSS −−> PFC −−> PTG	0.023	0.098	0.072 ***
PSS −−> EFC −−> PTG	0.007	0.042	0.046 *
E −−> EFC −−> PTG	0.093	0.478	0.060 **
A −−> AC−−> PTG	−0.368	−0.049	−0.045 *
C−−> AC−−> PTG	−0.433	−0.090	−0.052 **
N −−> PFC −−> PTG	0.010	0.421	0.040 ✝
A −−> EFC−−> PTG	0.002	0.362	0.033 ✝
E −−> PFC −−> PTG	−0.152	0.122	−0.002
E −−> AC −−> PTG	−0.202	0.064	−0.015
E −−> SSC −−> PTG	−0.042	0.031	0.000
A−−> PFC −−> PTG	−0.164	−0.164	−0.005
A −−> SSC −−> PTG	−0.05	0.037	0.000
C −−> PFC −−> PTG	−0.069	0.304	0.020
C −−> SSC −−> PTG	−0.045	0.046	0.000
C −−> EFC−−> PTG	−0.101	0.268	0.015
N −−>AC −−> PTG	−0.33	0.035	−0.029
N −−> SSC −−> PTG	−0.144	0.115	−0.001
N −−> EFC −−> PTG	−0.097	0.331	0.025
O −−> PFC −−> PTG	−0.17	0.229	0.005
O −−> AC −−> PTG	−0.187	0.168	−0.002
O −−>SSC −−> PTG	−0.079	0.049	0.000
O −−> EFC −−> PTG	−0.079	0.336	0.02
PSS −−> AC −−> PTG	−0.039	0.018	−0.011
PSS −−> SSC −−> PTG	−0.036	0.044	0.001

**Note.** PTG = Post-Traumatic Growth, PSS = Perceived Social Support, E = Extraversion, A = Agreeableness, C = Conscientiousness, N = Emotional Stability, O = Openness, PFC = Problem-focused Coping, EFC = Emotion-focused Coping, AC = Avoidance Coping, SSC = Social Support Coping. *** = *p* < 0.001, ** = *p* < 0.01, * = *p* < 0.05, ✝ = *p* < −0.1.

## References

[1] Caballero-Domínguez C.C., Jiménez-Villamizar M.P., Campo-Arias A. Suicide risk during the lockdown due to coronavirus disease (COVID-19) in Colombia. https://pubmed.ncbi.nlm.nih.gov/32589519/.

[2] Cheung T., Lam S.C., Lee P.H., Xiang Y.T., Yip P.S.F. (2021). Global Imperative of Suicidal Ideation in 10 Countries Amid the COVID-19 Pandemic. Front. Psychiatry.

[3] Finstad G.L., Giorgi G., Lulli L.G., Pandolfi C., Foti G., León-Perez J.M., Cantero-Sánchez F.J., Mucci N. (2021). Resilience, Coping Strategies and Posttraumatic Growth in the Workplace Following COVID-19: A Narrative Review on the Positive Aspects of Trauma. Int. J. Environ. Res. Public Health.

[4] Stallard P., Pereira A.I., Barros L. (2021). Post-traumatic growth during the COVID-19 pandemic in carers of children in Portugal and the UK: Cross-sectional online survey. BJPsych Open.

[5] Tedeschi R.G., Calhoun L.G. (1996). The Posttraumatic Growth Inventory: Measuring the positive legacy of trauma. J. Trauma. Stress.

[6] Tedeschi R.G., Calhoun L.G. (2004). TARGET ARTICLE:“Posttraumatic Growth: Conceptual Foundations and Empirical Evidence”. Psychol. Inq..

[7] Helgeson V.S., Reynolds K.A., Tomich P.L. (2006). A meta-analytic review of benefit finding and growth. J. Consult. Clin. Psychol..

[8] Tedeschi R.G., Shakespeare-Finch J., Taku K., Calhoun L.G. (2018). Posttraumatic Growth: Theory, Research, and Applications.

[9] Ng Q.X., Lim D.Y., Chee K.T. (2020). Not all trauma is the same. Proc. Natl. Acad. Sci. USA.

[10] Horesh D., Brown A.D. (2020). Traumatic stress in the age of COVID-19: A call to close critical gaps and adapt to new realities. Psychol. Trauma Theory Res. Pract. Policy.

[11] Le K., Nguyen M. (2020). The psychological consequences of COVID-19 lockdowns. Int. Rev. Appl. Econ..

[12] Casellas-Grau A., Ochoa C., Ruini C. (2017). Psychological and clinical correlates of posttraumatic growth in cancer: A systematic and critical review. Psycho-Oncol..

[13] Rzeszutek M., Gruszczyńska E. (2018). Posttraumatic growth among people living with HIV: A systematic review. J. Psychosom. Res..

[14] Garcia F.E., Cova F., Rincón P., Vazquez C. (2015). Trauma or growth after a natural disaster? The mediating role of rumination processes. Eur. J. Psychotraumatology.

[15] Chen R., Sun C., Chen J., Jen H., Kang X.L., Kao C., Chou K. (2020). A Large-Scale Survey on Trauma, Burnout, and Posttraumatic Growth among Nurses during the COVID-19 Pandemic. Int. J. Ment. Health Nurs..

[16] Cui P.P., Wang P.P., Wang K., Ping Z., Wang P., Chen C. (2020). Post-traumatic growth and influencing factors among frontline nurses fighting against COVID-19. Occup. Environ. Med..

[17] Edmondson A.C., Lei Z. (2014). Psychological Safety: The History, Renaissance, and Future of an Interpersonal Construct. Annu. Rev. Organ. Psychol. Organ. Behav..

[18] Galea S., Merchant R.M., Lurie N. (2020). The Mental Health Consequences of COVID-19 and Physical Distancing. JAMA Intern. Med..

[19] Vazquez C., Valiente C., García F.E., Contreras A., Peinado V., Trucharte A., Bentall R.P. (2021). Post-Traumatic Growth and Stress-Related Responses During the COVID-19 Pandemic in a National Representative Sample: The Role of Positive Core Beliefs About the World and Others. J. Happiness Stud..

[20] Durak E.S., Ayvasik H.B. (2010). Factors Associated with Posttraumatic Growth among Myocardial Infarction Patients: Perceived Social Support, Perception of the Event and Coping. J. Clin. Psychol. Med. Settings.

[21] Jia X., Ying L., Zhou X., Wu X., Lin C. (2015). The Effects of Extraversion, Social Support on the Posttraumatic Stress Disorder and Posttraumatic Growth of Adolescent Survivors of the Wenchuan Earthquake. PLoS ONE.

[22] Eagle D.E., Hybels C.F., Proeschold-Bell R.J. (2018). Perceived social support, received social support, and depression among clergy. J. Soc. Pers. Relatsh..

[23] Alisic E., Boeije H.R., Jongmans M.J., Kleber R.J. (2011). Children’s Perspectives on Dealing with Traumatic Events. J. Loss Trauma.

[24] Schroevers M.J., Helgeson V.S., Sanderman R., Ranchor A.V. (2010). Type of social support matters for prediction of posttraumatic growth among cancer survivors. Psycho-Oncol..

[25] Kilmer R.P., Gil-Rivas V., Griese B., Hardy S., Hafstad G.S., Alisic E. (2014). Posttraumatic growth in children and youth: Clinical implications of an emerging research literature. Am. J. Orthopsychiatry.

[26] Panjikidze M., Beelmann A., Martskvishvili K., Chitashvili M. (2019). Posttraumatic Growth, Personality Factors, and Social Support among War-Experienced Young Georgians. Psychol. Rep..

[27] Saltzman L.Y., Hansel T.C., Bordnick P.S. (2020). Loneliness, isolation, and social support factors in post-COVID-19 mental health. Psychol. Trauma Theory Res. Pract. Policy.

[28] Garnefski N., Kraaij V., Schroevers M.J., Somsen G.A. (2008). Post-Traumatic Growth after a Myocardial Infarction: A Matter of Personality, Psychological Health, or Cognitive Coping?. J. Clin. Psychol. Med. Settings.

[29] Mattson E., James L., Engdahl B. (2018). Personality Factors and Their Impact on PTSD and Post-traumatic Growth is Mediated by Coping Style Among OIF/OEF Veterans. Mil. Med..

[30] Măirean C. (2016). The Relationship Between Secondary Traumatic Stress and Personal Posttraumatic Growth: Personality Factors as Moderators. J. Adult Dev..

[31] Koliouli F., Canellopoulos L. (2021). Dispositional optimism, stress, post-traumatic stress disorder and post-traumatic growth in Greek general population facing the COVID-19 crisis. Eur. J. Trauma Dissociation.

[32] Prati G., Pietrantoni L. (2009). Optimism, Social Support, and Coping Strategies as Factors Contributing to Posttraumatic Growth: A Meta-Analysis. J. Loss Trauma.

[33] Jaarsma T.A., Pool G., Sanderman R., Ranchor A.V. (2006). Psychometric properties of the Dutch version of the posttraumatic growth inventory among cancer patients. Psycho-Oncol..

[34] Sheikh A.I. (2004). Posttraumatic Growth in the Context of Heart Disease. J. Clin. Psychol. Med. Settings.

[35] Zoellner T., Rabe S., Karl A., Maercker A. (2008). Posttraumatic growth in accident survivors: Openness and optimism as predictors of its constructive or illusory sides. J. Clin. Psychol..

[36] Tomita M., Takahashi M., Tagaya N., Kakuta M., Kai I., Muto T. (2016). Structural equation modeling of the relationship between posttraumatic growth and psychosocial factors in women with breast cancer. Psycho-Oncol..

[37] Sébille V., Hardouin J.-B., Giral M., Bonnaud-Antignac A., Tessier P., Papuchon E., Jobert A., Faurel-Paul E., Gentile S., Cassuto E. (2016). Prospective, multicenter, controlled study of quality of life, psychological adjustment process and medical outcomes of patients receiving a preemptive kidney transplant compared to a similar population of recipients after a dialysis period of less than three years—The PreKit-QoL study protocol. BMC Nephrol..

[38] Tuncay T., Musabak I. (2015). Problem-Focused Coping Strategies Predict Posttraumatic Growth in Veterans with Lower-Limb Amputations. J. Soc. Serv. Res..

[39] Stutts L.A., Bills S.E., Erwin S.R., Good J.J. (2015). Coping and posttraumatic growth in women with limb amputations. Psychol. Health Med..

[40] Larsen S.E., Berenbaum H. (2015). Are Specific Emotion Regulation Strategies Differentially Associated with Posttraumatic Growth Versus Stress?. J. Aggress. Maltreatment Trauma.

[41] Peng A.C., Riolli L.T., Schaubroeck J., Spain E.S.P. (2011). A moderated mediation test of personality, coping, and health among deployed soldiers. J. Organ. Behav..

[42] He L., Xu J., Wu Z. (2013). Coping Strategies as a Mediator of Posttraumatic Growth among Adult Survivors of the Wenchuan Earthquake. PLoS ONE.

[43] London M.J., Mercer M.C., Lilly M.M. (2017). Considering the Impact of Early Trauma on Coping and Pathology to Predict Posttraumatic Growth among 9-1-1 Telecommunicators. J. Interpers. Violence.

[44] Brooks M., Graham-Kevan N., Robinson S., Lowe M. (2019). Trauma characteristics and posttraumatic growth: The mediating role of avoidance coping, intrusive thoughts, and social support. Psychol. Trauma Theory Res. Pr. Policy.

[45] Hagenaars M.A., Fisch I., van Minnen A. (2011). The effect of trauma onset and frequency on PTSD-associated symptoms. J. Affect. Disord..

[46] Scrignaro M., Barni S., Magrin M.E. (2010). The combined contribution of social support and coping strategies in predicting post-traumatic growth: A longitudinal study on cancer patients. Psycho-Oncol..

[47] Cole A.S., Lynn S.J. (2010). Adjustment of Sexual Assault Survivors: Hardiness and Acceptance Coping in Posttraumatic Growth. Imagin. Cogn. Pers..

[48] Kato T. (2012). Development of the Coping Flexibility Scale: Evidence for the Coping Flexibility Hypothesis. J. Couns. Psychol..

[49] Büyükaşik-Çolak C., Aktürk E.G., Bozo Ö. (2012). Mediating Role of Coping in the Dispositional Optimism–Posttraumatic Growth Relation in Breast Cancer Patients. J. Psychol..

[50] Bellur Z., Aydın A., Alpay E.H. (2018). Mediating role of coping styles in personal, environmental and event related factors and posttraumatic growth relationships in women with breast cancer. Klin. Psikiyatr. Derg..

[51] Yu Y., Peng L., Chen L., Long L., He W., Li M., Wang T. (2014). Resilience and social support promote posttraumatic growth of women with infertility: The mediating role of positive coping. Psychiatry Res..

[52] Shakespeare-Finch J., Gow K., Smith S. (2005). Personality, Coping and Posttraumatic Growth in Emergency Ambulance Personnel. Traumatology.

[53] Connor-Smith J.K., Flachsbart C. (2007). Relations between personality and coping: A meta-analysis. J. Pers. Soc. Psychol..

[54] Margetić B., Peraica T., Stojanović K., Ivanec D. (2021). Predictors of emotional distress during the COVID-19 pandemic; a Croatian study. Pers. Individ. Differ..

[55] Bartley C.E., Roesch S.C. (2011). Coping with daily stress: The role of conscientiousness. Pers. Individ. Differ..

[56] Gori A., Topino E., Sette A., Cramer H. (2020). Pathways to post-traumatic growth in cancer patients: Moderated mediation and single mediation analyses with resilience, personality, and coping strategies. J. Affect. Disord..

[57] Zimet G.D., Dahlem N.W., Zimet S.G., Farley G.K. (1988). The Multidimensional Scale of Perceived Social Support. J. Persinal. Assess..

[58] Gosling S.D., Rentfrow P.J., Swann W.B. (2003). A very brief measure of the Big-Five personality domains. J. Res. Pers..

[59] Costa P.T., McCrae R.R. (1992). Normal personality assessment in clinical practice: The NEO Personality Inventory. Psychol. Assess..

[60] Carver C.S. (1997). You want to measure coping but your protocol’s too long: Consider the Brief COPE. Int. J. Behav. Med..

[61] Bose C.N., Bjorling G., Elfstrom M.L., Persson H., Saboonchi F. (2015). Assessment of Coping Strategies and Their Associations with Health Related Quality of Life in Patients with Chronic Heart Failure: The Brief COPE Restructured. Cardiol. Res..

[62] Schermelleh-Engel K., Moosbrugger H., Müller H. (2003). Evaluating the fit of structural equation models: Tests of significance and descriptive goodness-of-fit measures. Methods Psychol. Res. Online.

[63] Moshagen M., Erdfelder E. (2016). A New Strategy for Testing Structural Equation Models. Struct. Equ. Modeling: A Multidiscip. J..

[64] Kroencke L., Geukes K., Utesch T., Kuper N., Back M. (2020). Neuroticism and emotional risk during the COVID-19 pandemic. J. Res. Pers..

[65] Zoellner T., Maercker A. (2006). Posttraumatic growth in clinical psychology—A critical review and introduction of a two component model. Clin. Psychol. Rev..

[66] Shigemoto Y., Poyrazli S. (2013). Factors related to posttraumatic growth in US and Japanese college students. Psychol. Trauma Theory Res. Pr. Policy.

[67] Cervone D., Pervin L.A. (2013). Personality: Theory and Research.

[68] Harris G.M., Allen R.S., Dunn L., Parmelee P. (2013). “Trouble won’t last always” religious coping and meaning in the stress process. Qual. Health Res..

[69] Wu K., Zhang Y., Liu Z., Zhou P., Wei C. (2015). Coexistence and different determinants of posttraumatic stress disorder and posttraumatic growth among Chinese survivors after earthquake: Role of resilience and rumination. Front. Psychol..

[70] Yang Y., Sun G., Dong X., Zhang H., Xing C., Liu Y. (2019). Preoperative anxiety in Chinese colorectal cancer patients: The role of social support, self-esteem and coping styles. J. Psychosom. Res..

[71] Waugh C.E., Shing E.Z., Furr R.M. (2020). Not all disengagement coping strategies are created equal: Positive distraction, but not avoidance, can be an adaptive coping strategy for chronic life stressors. Anxiety Stress Coping.

[72] Maercker A., Zoellner T. (2004). The Janus face of self-perceived growth: Toward a two-component model of posttraumatic growth. Psychol. Inq..

[73] Trobst K.K. (2000). An Interpersonal Conceptualization and Quantification of Social Support Transactions. Pers. Soc. Psychol. Bull..

[74] Lee J., Loke W., Ng Q. (2020). The Role of Family Physicians in a Pandemic: A Blueprint. Healthcare.

[75] Schafer K.M., Lieberman A., Sever A.C., Joiner T. (2021). Prevalence rates of anxiety, depressive, and eating pathology symptoms between the pre- and peri-COVID-19 eras: A meta-analysis. J. Affect. Disord..

[76] Prieto-Ursúa M., Jódar R. (2020). Finding Meaning in Hell. The Role of Meaning, Religiosity and Spirituality in Posttraumatic Growth During the Coronavirus Crisis in Spain. Front. Psychol..

[77] Krägeloh C., Chai P.P.M., Shepherd D., Billington R. (2010). How Religious Coping is Used Relative to Other Coping Strategies Depends on the Individual’s Level of Religiosity and Spirituality. J. Relig. Health.

[78] Kashyap S., Hussain D. (2017). Cross-Cultural Challenges to the Construct “Posttraumatic Growth”. J. Loss Trauma.

[79] Earley P.C. (1989). Social Loafing and Collectivism: A Comparison of the United States and the People’s Republic of China. Adm. Sci. Q..

